# Molecular diagnosis of patients with hepatitis A virus infection using amplicon-based nanopore sequencing

**DOI:** 10.1371/journal.pone.0288361

**Published:** 2023-07-12

**Authors:** Geum-Young Lee, Kyungmin Park, Young-Sun Lee, Ji Hoon Kim, Kwan Soo Byun, Jongwoo Kim, Won-Keun Kim, Jin-Won Song

**Affiliations:** 1 Department of Microbiology, Korea University College of Medicine, Seoul, Republic of Korea; 2 Department of Biomedical Sciences, BK21 Graduate Program, Korea University College of Medicine, Seoul, Republic of Korea; 3 Department of Internal Medicine, Division of Gastroenterology and Hepatology, Korea University Medical Center, Seoul, Republic of Korea; 4 Department of Microbiology, College of Medicine, Hallym University, Chuncheon, Republic of Korea; 5 Institute of Medical Research, College of Medicine, Hallym University, Chuncheon, Republic of Korea; Shanghai Public Health Clinical Center, Fudan University, CHINA

## Abstract

High-throughput sequencing is a robust tool used for identifying and tracking pathogen outbreaks. Whole-genome sequencing of hepatitis A virus (HAV) remains poor due to ultra-low viral loads, limitations of next-generation sequencing technology, and its high costs in clinical applications. This study evaluated multiplex polymerase chain reaction (PCR)-based nanopore sequencing to obtain whole-genome sequences of HAV. The HAV genomes were obtained directly from patient specimens for a rapid molecular diagnosis of viral genotypes. Serum and stool samples were collected from six patients with hepatitis A infection. Amplicon-based nanopore sequencing was performed from the clinical specimens to identify HAV genotypes by acquiring nearly complete-genome sequences. TaqMan-based quantitative PCR (qPCR) was conducted to detect and quantify multiple HAV genes. Singleplex-based nanopore sequencing demonstrated high genome coverage rates (90.4–99.5%) of HAV within 8 h, at viral RNA loads of 10 to 10^5^ copies/μL. TaqMan qPCR showed multiplex quantification of HAV genes namely, VP0, VP3, and 3C. This study provides useful insights into rapid molecular diagnosis during hepatitis A outbreaks and may ultimately augment public health disease surveillance in the hospital and epidemiology field.

## Introduction

Hepatitis A virus (HAV) is a non-enveloped positive-stranded RNA virus. It belongs to the genus *Hepatovirus* in the family *Picornaviridae* and poses a critical public health threat [1]. HAV genome consists of a large open reading frame, including the P1 (VP1, VP2, VP3, and VP4), P2 (2A, 2 B, and 2C), and P3 (3A, 3 B, 3C, and 3D) regions. Human HAV strains include four genotypes (I–III and VII) and six sub-genotypes (IA, IB, IIA, IIB, IIIA, and IIIB). The virus spreads via the fecal-oral route from contaminated foods and water. Hepatitis A occurs sporadically and epidemically, with a tendency toward cyclic recurrence [2]. In the Republic of Korea (ROK), a large HAV outbreak of 17,635 cases was reported in 2019, which represents the highest incidence since 2009.

Next-generation sequencing (NGS) has proven to be a robust platform for identifying and characterizing pathogens to investigate population demographics, etiologic agents, and outbreak locations [3–6]. The MinION sequencer, developed by Oxford Nanopore Technologies (ONT), is a palm-sized portable device that is smaller and cheaper than the traditional NGS platform [7,8]. The nanopore system enables real-time sequencing, which is advantageous for rapid molecular diagnoses of zoonotic pathogens such as severe acute respiratory syndrome coronavirus 2 (SARS-CoV-2) and Hantaan virus, in hospitals and fields [9,10]. The nanopore sequencing identified the causative serotype of the foot-and-mouth disease virus within a minimum time (5 h) [11]. Recently, whole-genome sequencing of HAV was performed from an isolate using a polymerase chain reaction-free (PCR)-free single-molecule nanopore sequencing approach [12]. However, whole-genome sequencing of HAV from clinical samples remains to be developed using nanopore-based NGS.

In this study, we established a multiplex PCR-based nanopore sequencing assays for nearly whole-genome sequencing of HAV from clinical samples. These findings provide valuable insights into rapid sequencing and molecular diagnosis during hepatitis A outbreaks and may ultimately augment public health disease surveillance in the hospital and epidemiology field.

## Materials & methods

### Ethical approval and informed consent

Patient samples were provided from the Korea University Guro Hospital, Seoul, ROK. Written informed consent was acquired from all participants. This study was approved by the Institutional Review Board of Korea University Guro Hospital (2019GR0417). All methods were conducted in accordance with relevant guidelines and regulations.

### Sample collection and RNA extraction

Six patients with hepatitis A infection were diagnosed on the basis of clinical and laboratory tests. Sera and stool samples were collected from patients aged 22–58 years. Total RNA was extracted from the sera and stool specimens using TRI Reagent Solution LS (Ambion, Austin, TX, USA), according to the manufacturer’s instructions.

### TaqMan quantitative PCR (qPCR) assay

cDNA was synthesized from 1 μg of RNA using a high-capacity RNA-to-cDNA kit (Applied Biosystems, Foster City, CA, USA) and random hexamers. qPCR was performed using the TaqMan Multiplex Master Mix (Applied Biosystems) on a Quantstudio 5 Flex Real-time PCR System (Applied Biosystems). The composition of 25 μL of the reaction mixture was 10 μL of TaqMan PCR Multiplex Master Mix (Applied Biosystems), 1 μL of cDNA template, 10 pM of each TaqMan probe, 18 pM of each forward and reverse direction primer, and 7 μL of D.W. The PCR conditions were 95°C for 10 min, followed by 45 cycles of 95°C for 15 s, 56°C for 1 min, and 65°C for 1 min. The cutoff value is 40. The sequences of each probe and primer used in this study are shown in S1 Table.

### Quantitation of HAV genome copy numbers

Recombinant plasmid DNA of the HAV VP3 gene was isolated using the pTOP Blunt V2 vector (Enzynomics, Seoul, ROK). The concentration of recombinant plasmid DNA was evaluated by measuring UV absorbance at 260 and 280 nm using a Nanodrop spectrophotometer (Thermo Fisher Scientific, CA, USA). Serial dilutions of recombinant plasmid DNA standards ranging from 10 to 10^10^ copies/μL were amplified using the TaqMan PCR Master Mix (Applied Biosystems) on a Quantstudio 5 Flex Real-time PCR System (Applied Biosystems). Primer sequences and PCR conditions were identical to those used for the TaqMan qPCR assay above. The copy number of plasmids per μg of DNA was determined from the total number of nucleotides using a formula described previously [13]. Each data point represents the mean cycle threshold (Ct) value obtained from triplicate experiments.

### Multiplex PCR

Multiplex PCR primers were designed for complete genome sequencing of HAV genomes (S2 Table). cDNA template was amplified using Solg 2× Uh-Taq PCR Smart Mix (SolGent, Seoul, ROK) according to the manufacturer’s instructions. PCR reaction mixture contained 12.5 μL of 2× Uh pre-mix, 1 μL of cDNA template, 1 pM of each primer (final concentration: 0.04 pM), 9.5 μL of distilled water in a final volume of 25 μL. The first and second round PCR was conducted at 95°C for 15 min, followed by 40 and 25 cycles at 95°C for 20 s, 50°C for 40 s, 72°C for 1 min, and final elongation at 72°C for 3 min, respectively.

### Nanopore sequencing for HAV

**Fig 1:**
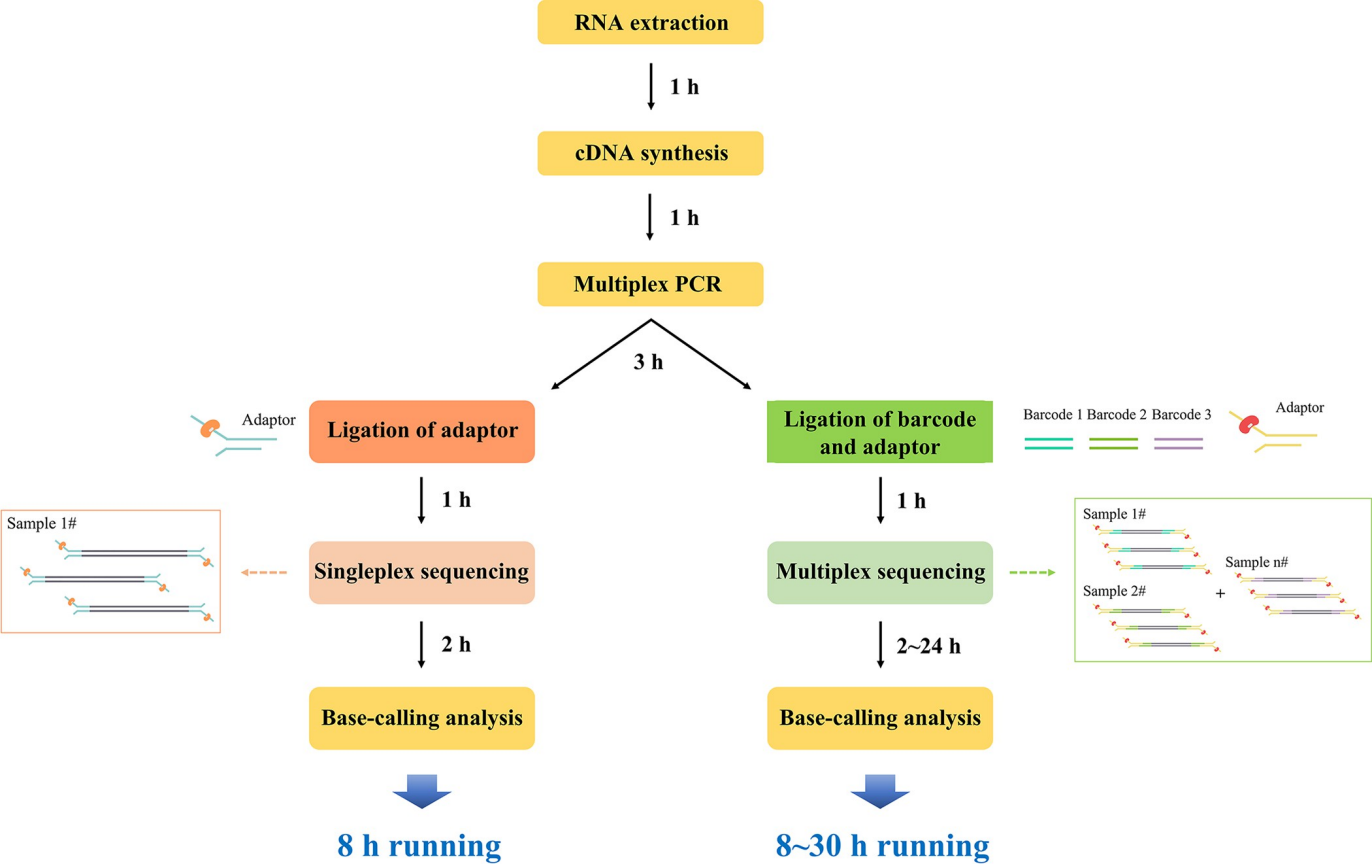
Workflow overview of singleplex and multiplex-based nanopore sequencing for hepatitis A virus (HAV). The singleplex assay (single specimen test) was performed in 8 h with 2 h sequencing time. The multiplex assay (barcode indexing method) was conducted in 8–30 h with 2–24 h sequencing time, respectively.

The DNA library was prepared using a Ligation Sequencing Kit (SQK-LSK109; ONT, London, UK) with a Native Barcoding Kit (EXP-NBD104; ONT) according to the manufacturer’s instructions. The library was sequenced on an MK1C device (ONT) with an FLO-MIN-106 flow cell (v9.4.1). Each sample library was sequenced using a singleplex assay for 2 h. Barcoded multiplex libraries were analyzed on Mk1C for 2–24 h of sequencing time. Libraries were purified on a magnetic rack and cleaned up using AMPure XP beads (Beckman Coulter, Brea, CA, USA). Real-time basecalling and sequence trimming were performed using Guppy (v3.0.3) on the MK1C device. Total reads were trimmed to remove the adaptor and index sequences, and filtered only over the reads (with a Q-score > 7) for the subsequent analysis to improve the data reliability during real-time basecalling using Guppy (v3.0.3). The filtered sequences were combined into a single FASTA file using the Porechop (v.9.0). The consensus sequences were extracted using CLC Genomics Workbench (v7.5.2; Qiagen, Hilden, Germany). The reads mapped to the reference sequence (LU38 strain) and calculated using the following parameters: initial trimming of read length under 50 and over 7 kb reads, discarding both 5’ and 3’ end 10 sequences, read aliment match score 2, mismatch cost 3, length fraction 0.7, similarity fraction 0.8, and ignore of non-specific match handling. The uncovered genomic sequences of HAV were determined by conventional RT-PCR. The workflow of nanopore sequencing in this study is shown in Fig 1.

### Illumina sequencing for HAV

The libraries were prepared using the TruSeq Nano DNA HT sample preparation kit (Illumina, San Diego, USA) according to the manufacturer’s instructions. NGS was performed on a MiSeq benchtop sequencer platform (Illumina) with 2 × 150 bp by using a MiSeq reagent kit v2 (Illumina). Total reads were trimmed to the adaptor and index sequences, and low-quality reads (lower than Q20) were filtered using CLC Genomics Workbench (v7.5.2; Qiagen). The filtered reads were mapped to the HAV LU38 strain, and consensus sequences were extracted in accordance with the above parameters used in the nanopore sequencing. The uncovered genomic sequences of HAV were determined by conventional RT-PCR.

### Phylogenetic analysis

The HAV genomic sequences were aligned using ClustalW in Lasergene 5 (DNASTAR, Madison, WI, USA). A phylogenetic tree was generated using the best-fit GTR+G+I model of evolution using the maximum-likelihood method. The pairwise genetic distance between the HAV strains was calculated using MEGA 7.0 [14]. Support for topologies was assessed using bootstrapping for 1,000 iterations.

## Results

### Clinical and laboratory diagnosis of patients with HAV infection

Six patients exhibited symptoms of acute hepatitis A infection (1). All patients positive for anti-HAV IgM antibodies showed significant elevation in aspartate aminotransferase, alanine aminotransferase, and alkaline phosphatase levels. Four patients manifested serum bilirubin levels > 1.2 mg/dl. HAV KUMC 19–1 showed hypoalbuminemia with serum albumin levels of < 3.5 g/dl. C-reactive protein (CRP) levels and white blood cell (WBC) counts significantly increased in all patients who manifested typical symptoms compatible with acute viral hepatitis A. Fever, fatigue, myalgia, nausea/vomiting, abdominal discomfort, headache, jaundice, and diarrhea were observed in 100% (6/6), 50% (3/6), 50% (3/6), 50% (3/6), 50% (3/6), 16.7% (1/6), 66.7% (4/6), and 0% (0/6) of patients, respectively.

**Table 1:** Characteristics and laboratory test results of patients with acute hepatitis A infections in the Republic of Korea.

	KUMC 19–1	KUMC 20–1	KUMC 20–2	KUMC 20–3	KUMC 20–4	KUMC 20–5
**Demographics**						
Age	41	39	43	22	58	28
Sex	Female	Male	Male	Female	Female	Female
Anti-HAV IgM (s/c)	1.32 COI	13.64 COI	14.36 COI	18 COI	8.47 COI	6.88 COI
**Laboratory tests**						
AST (IU/L)	1041	91	2833	2463	1992	437
ALT (IU/L)	719	360	4199	1390	4448	1495
ALP (IU/L)	103	129	370	200	238	223
Total bilirubin (mg/dl)	1.15	17.9	7.72	1.21	4.68	3.18
Prothrombin time (%)	102	104	90	90	68	115
Albumin (g/ml)	3.2	3.8	4.2	4.2	4	4.3
Hemoglobin (g/dl)	12.7	16.9	14.7	14.1	14.4	14.8
Creatinine (mg/dL)	0.46	0.51	0.51	0.68	0.68	0.48
CRP (mg/dL)	25.8	17.48	17.35	34.69	36.3	17.45
WBC (10^9^/L)	2	5.4	6.8	1.8	6.4	5.4
Platelets (10^9^/L)	89	251	186	120	160	191
**Symptoms**						
Fever	YES	YES	YES	YES	YES	YES
Fatigue	NO	YES	NO	NO	YES	YES
Myalgia	NO	YES	NO	NO	YES	YES
Nausea/vomiting	YES	NO	YES	YES	NO	NO
Abdominal discomfort	NO	NO	YES	NO	YES	YES
Headache	NO	NO	NO	YES	NO	NO
Jaundice	NO	YES	YES	NO	YES	YES
Diarrhea	NO	NO	NO	NO	NO	NO

ALT, alanine aminotransferase; AST, aspartate aminotransferase; ALP, alkaline phosphatase; anti-IgM, anti-immunoglobulin M; anti-IgG, anti-immunoglobulin G; CRP, C-reactive protein; HAV, hepatitis A virus; WBC, white blood cell.

### Quantification of HAV genomes

**Fig 2:**
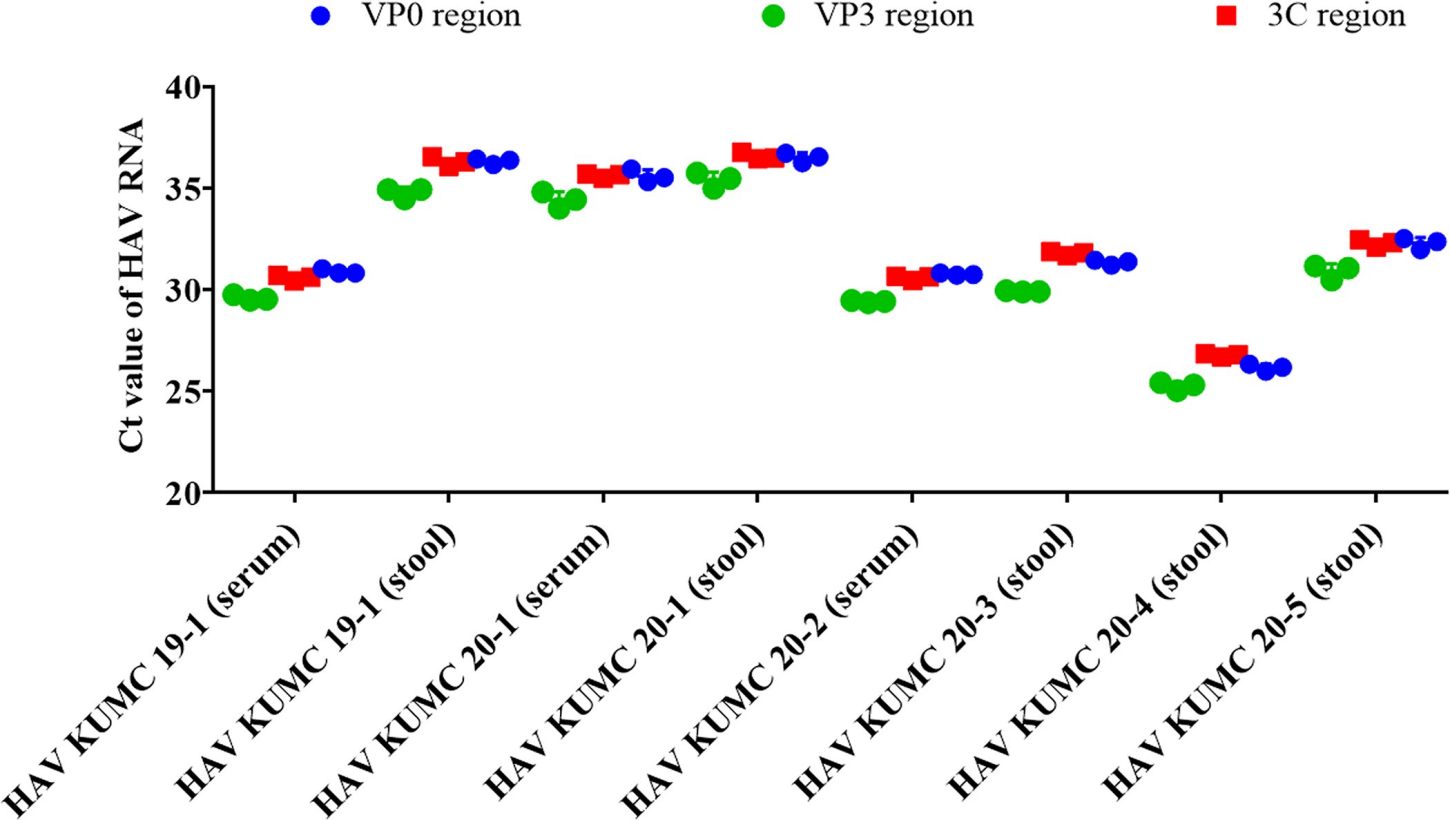
Measurement for HAV RNA from eight clinical samples. Diagnosis and quantification of HAV genomes in serum and stool samples from six patients were performed using the real-time multiplex TaqMan-based qPCR assays. Ct values were determined for the HAV RNA of three regions (VP0, VP3, and 3C genes). HAV, Hepatitis A virus; Ct, cycle threshold; qPCR, quantitative polymerase-chain reaction.

HAV RNA loads were quantified using VP0 (VP4/VP2), VP3, and 3C-specific TaqMan qPCR (Fig 2). HAV KUMC 19–1 (serum), HAV KUMC 20–2 (serum), HAV KUMC 20–3 (stool), HAV KUMC 20–4 (stool), and HAV KUMC 20–5 (stool) showed Ct values of 26.1 to 31.8, corresponding to the viral load of 2.4–4.5 log_10_ copies/μL. The Ct values of HAV KUMC 19–1 (stool), HAV KUMC 20–1 (serum), and HAV KUMC 20–1 (stool) ranged from 35.2–36.2, corresponding to the viral RNA of 1.4–1.7 log_10_ copies/μL.

### Whole-genome sequencing of HAV from clinical specimens

Using singleplex nanopore sequencing assay, nearly complete genome sequences of HAV (95.6–99.5%) were recovered from the five samples of patients who possessed 10^2^–10^5^ copies/μL of viral RNA (Table 2). Three clinical specimens showed relatively lower coverage rates ranged 90.4–94.8% because of the lowest amount of HAV genomes, corresponding to 10–10^2^ copies/μL of viral RNA. A total of 114,705 to 788,945 reads were mapped to the reference sequence of HAV, and the mean depth of coverage was 6,087 to 50,192.

**Table 2:** Genome coverage and mean depth for hepatitis A virus (HAV) using singleplex-based nanopore sequencing.

HAV RNA copy number (copies/μL)	Sample	Type	Coverage rate (%) ^a^	Total Reads	Reads mapped to reference sequence ^a^	Rate of reads mapped	Depth of coverage ^b^
10^4^−10^5^	KUMC 20–4	Stool	99.5	375,429	238,170	63.4	10,854
10^2^−10^3^	KUMC 20–2	Serum	96.5	923,505	738,042	79.9	43,431
	KUMC 19–1	Serum	95.6	921,252	788,945	85.6	50,192
	KUMC 20–3	Stool	95.8	510,007	265,166	52.0	16,314
	KUMC 20–5	Stool	97	1,164,894	161,392	13.9	6,593
10−10^2^	KUMC 20–1	Serum	94.8	992,353	231,318	23.3	16,855
	KUMC 19–1	Stool	90.9	840,597	427,373	50.8	21,793
	KUMC 20–1	Stool	90.4	270,055	114,705	42.5	6,087

^a^; Genome coverage rates and viral reads mapped to a reference sequence were calculated using the LU38 strain.

^b^; Depth of coverage was calculated by the number of mapped reads (read length × number of reads matching the reference/reference genome size).

The native barcoding-based nanopore sequencing method was used to analyze the HAV genome from multiple samples within 24 h. HAV genome sequences of 68.3–82.6% were recovered from clinical specimens, including 10^2^–10^5^ copies/μL of HAV RNA (S1 Fig and S3 Table). The clinical samples containing viral RNA loads of 10–10^2^ copies/μL revealed low coverage rates ranged from 34.3–49.8%. A total of 2,611 to 45,829 reads were mapped to the reference sequence of HAV, and the mean depth of coverage was 139 to 2,060.

The consensus accuracy of HAV genomic sequences was 99.41–99.79%, as compared to that generated by Illumina MiSeq system (S4 Table). The nucleotide insertion and deletion error rates of HAV genome sequences ranged from 0.04–0.1%.

### Phylogenetic analysis of HAV

**Fig 3:**
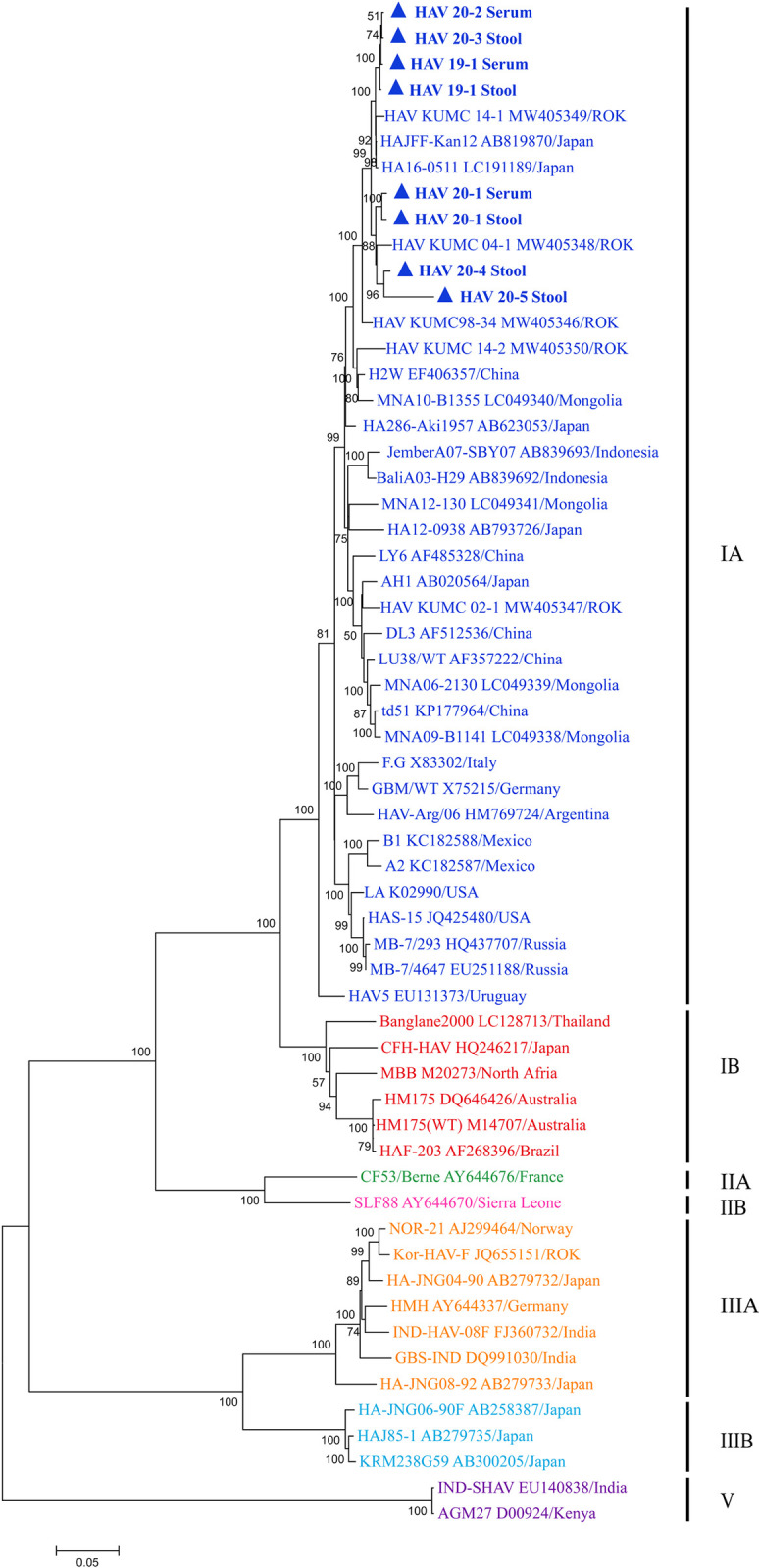
Phylogenetic analysis for nearly whole-genome sequences of hepatitis A virus (HAV) from hepatitis patients in this study. The used genomic sequences of HAV were obtained from serum and stool samples using singleplex-based nanopore sequencing. The phylogenetic tree was generated by the maximum likelihood method with 1,000 iterations. The colored circles indicate specific HAV strains from the ROK; blue, genotype IA; red, genotype IB; green, genotype IIA; pink, genotype IIB; orange, genotype IIIA; sky blue, genotype IIIB; and violet, genotype V, respectively.

A total of eight HAV genomes acquired in this study were genetically grouped with sub-genotype IA strains from ROK and Japan (Fig 3). The phylogenies of HAV 19–1 (serum), HAV 19–1 (stool), HAV 20–2 (serum), and HAV 20–3 (stool) formed a homologous genetic lineage with the HAV KUMC 14–1, HAJFF-Kan12, and HA16-0511 strains from ROK and Japan. Phylogenetically, HAV 20–1 (serum), HAV 20–1 (stool), HAV KUMC 20–4 (stool), and HAV KUMC 20–5 (stool) shared a common ancestor with HAV KUMC 04–1 in the ROK.

## Discussion

Approximately, 600 million cases of foodborne diseases have been reported, with 420,000 fatal cases occurring annually worldwide [15]. Viral food poisoning is commonly caused by the intake of contaminated water and food such as sandwiches, fruits, uncooked vegetables, and shellfish [16–18]. Whole-genome sequencing allows the identification of the transmission chains of foodborne strains and potential intervention points for viral outbreaks [19]. Recently, point-of-care (POC) genomic sequencing is being rapidly developed for foodborne diseases. The reasonable and reliable diagnoses with on-site application have considerable advantages over lab-based testing which requires expensive and inaccessible on-site equipment [20]. Moreover, POC technology can prevent, or greatly reduce, the spread of viruses by providing immediate information regarding food and patient conditions for further testing. However, complete genome sequencing of HAV from clinical samples is limited by the high cost of laboratory tests and time-consuming operations. This study aimed to propose whole-genome sequencing to expand the scope of HAV surveillance in the hospital and epidemiology field through real-time virus identification from patients.

The nanopore platform facilitates the real-time identification of infectious agents, making it particularly attractive for POC testing [21]. This deployable system has offered the application of real-time metagenomic sequencing in POC testing for virus outbreaks, including chikungunya, Ebola, and hepatitis C viruses in human blood samples [22]. Whole-genome sequencing of zoonotic pathogens in real time has contributed to the identification and characterization of an emerging virus outbreak using epidemiology with genotypic diversity, geographical origin, and infectious source [23–27]. Herein, multiplex PCR-based nanopore sequencing facilitated the nearly complete genome sequencing of HAV directly from human samples containing low copy numbers of viral RNA within 8 h using the singleplex assay. The accuracy of the HAV consensus sequences from the nanopore platform revealed 99.41–99.79% identity compared to the reference sequences from the Illumina MiSeq system. Our results suggest that amplicon-based nanopore sequencing may be useful for POC testing for real-time whole-genome sequencing of HAV in clinical cases.

qPCR assays are routinely used in clinical laboratories to determine the molecular etiology of patients [28,29]. Multiplex TaqMan qPCR has been useful for the simultaneous detection of multiple viruses or genes in a single reaction [30,31]. This assay offers significant cost- and time-saving potential for the accurate detection of newly emerging viruses [32,33]. In this study, TaqMan qPCR was developed to target multiple genes such as the VP0, VP3, and 3C of HAV. This approach provided the rapid and accurate monitoring of hepatitis A infections from clinical specimens with 10–10^5^ viral copies per μL. The sensitivity and accuracy of this assay can be improved by further large-scale surveillance. These findings suggest that multiplex TaqMan qPCR could be a rapid diagnostic method for investigating HAV outbreaks.

In this study, we had a limitation due to the small number of clinical samples. We were able to include eight specimens (sera and stools) from six patients with hepatitis A. Using serum samples, the laboratory test elucidated the clinical features of patients, containing AST, ALT, ALP, WBC, and platelet-related biomarkers. HAV genomes were detectable and recovered from the stools of hepatitis A patients. Our further studies should conduct a large-scale investigation of the specificity and sensitivity of the assay from additional clinical samples.

In conclusion, singleplex amplicon-based nanopore sequencing facilitated nearly whole-genome sequencing of HAV within 8 h. These findings demonstrate the potential application of nanopore technology for the rapid analysis of HAV in clinical specimens at a hospital and diagnosis field. The multiplex TaqMan qPCR assay was developed for the simultaneous detection of multiple HAV genes to monitor hepatitis A infection. This study provides significant insights into real-time whole-genome sequencing and molecular diagnosis for disease control, and mitigation strategies for emerging HAV outbreaks.

## Supporting information

S1 Fig Genome coverage rates for hepatitis A virus (HAV) obtained from nanopore sequencing at different time-points. The data shows the coverage rates of HAV genomes generated from multiplex PCR-based nanopore sequencing for 24 h running times using the native barcoded library assay.
(TIF)

S1 Table TaqMan probe and primer sequences for hepatitis A virus in this study. (PDF)

S2 Table Multiplex PCR primers for complete-genome sequencing of HAV genomes in this study. (PDF)

S3 Table Genome coverage rate and mean depth for hepatitis A virus (HAV) using nanopore sequencing in the multiplex assay for 24 h. (PDF)

S4 Table Indel error rate and consensus accuracy of the nanopore sequencing for hepatitis A virus (HAV) compared to Illumina MiSeq platform. (PDF)
