# Supplementary figures and images for: Molecular diagnosis of patients with hepatitis A virus infection using amplicon-based nanopore sequencing
Source: PLoS One. 2023 Jul 12;18(7):e0288361. doi: 10.1371/journal.pone.0288361 (PMC10337952; doi:10.1371/journal.pone.0288361)

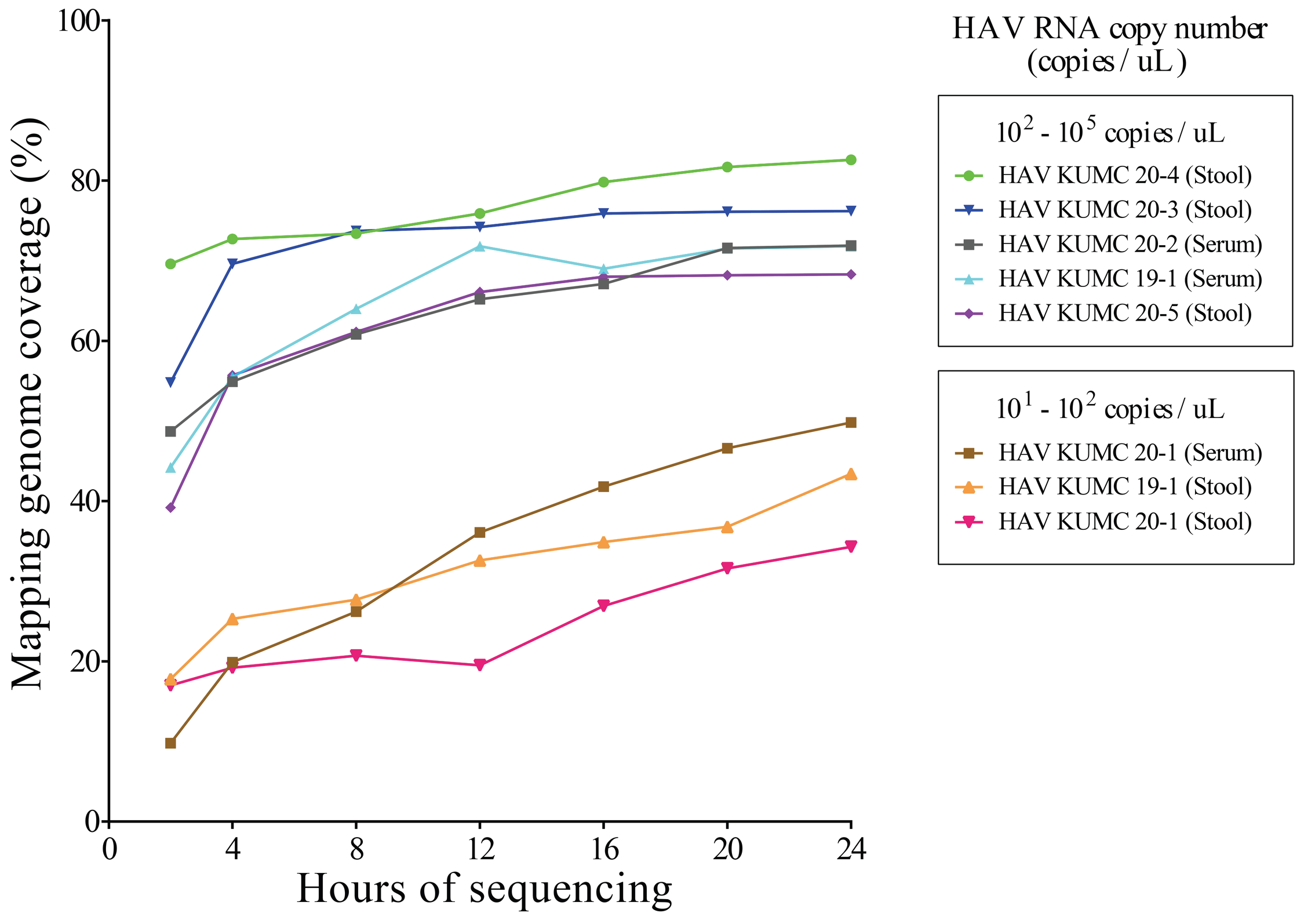

Supplement: S1 Fig — The data shows the coverage rates of HAV genomes generated from multiplex PCR-based nanopore sequencing for 24 h running times using the native barcoded library assay. (TIF) [file pone.0288361.s001.tif]
