# Supplementary material for: Molecular diagnosis of patients with hepatitis A virus infection using amplicon-based nanopore sequencing
Source: PLoS One. 2023 Jul 12;18(7):e0288361. doi: 10.1371/journal.pone.0288361 (PMC10337952; doi:10.1371/journal.pone.0288361)
Supplement: S1 Table — (PDF) [file pone.0288361.s002.pdf]

S1 Table. TaqMan probe and primer sequences for hepatitis A virus in this study.

Oligo	Name	Sequence (5′ to 3′)	5’ Reporter	3’ Quencher	Region	Size
Probe	VP0-P	AGA CTC AGG GGG AGA AG	VIC	MGBNFQ	960-979	
Forward primer	VP0-PF	TGA AAA CCT CTG TTG ATA AAC CTG			930-953	76 bp
Reverse primer	VP0-PR	CAA TCA GCA GAA TGA ATC AGG A			984-1005	
Probe	VP3-P	TGA CTT CTC CTT CTA ATG TTG CTT CTC A	FAM	MGBNFQ	2097-2124	
Forward primer	VP3-PF	GCC ATT GGG AAG CTT ATT GTG			2060-2080	107 bp
Reverse primer	VP3-PR	CAT TCC AAA TTA ATT GCT GAA AG			2144-2166	
Probe	3C-P	TGT GTG GTG GGG CCC TGG TGT C	ABY	QSY	5802-5823	
Forward primer	3C-PF	GGT CTT CCT GGA ATG TGT GG			5789-5808	67 bp
Reverse primer	3C-PR	CCA AAA TTG CAT TCT GTA TGG AC			5833-5855	
