# Supplementary material for: Molecular diagnosis of patients with hepatitis A virus infection using amplicon-based nanopore sequencing
Source: PLoS One. 2023 Jul 12;18(7):e0288361. doi: 10.1371/journal.pone.0288361 (PMC10337952; doi:10.1371/journal.pone.0288361)
Supplement: S2 Table — (PDF) [file pone.0288361.s003.pdf]

S2 Table. Multiplex PCR primers for complete-genome sequencing of HAV genomes in this study.

Primer	Sequence (5' to 3')	Position (nt)
HAV-1F	TTCAAGAGGGGTCTCCGGA	1-19
HAV-84F	AGGCTATAGGCTAAATTTCCCT	84-105
HAV-130R	AAGGAAATAGGGRAAGGAC	112-130
HAV-145R	TTACAACAAAACAAAAGGA	126-145
HAV-167R	CTGAACCTGCAGGAATTAATA	147-167
HAV-208F	ACGCTTTCTGTCTCYTTTCTT	208-228
HAV-212R	AGCGTGAAAATTGAGTGTTC	193-212
HAV-311R	GACTCCTACAGCTCCATGC	293-311
HAV-372R	GGT TCA TGA AAG CCA AGT TA	353-372
HAV-467R	TATCCGCCGCTGTTACCCTATCC	445-467
HAV-493F	CATTCAACGCCGGAGGACT	493-511
HAV-535R	CATTTAAGGCCAAATAGTGTTTG	513-535
HAV-564R	CTAGAGACAGCCCTGACAATC	554-564
HAV-595R	TGCCCTAAGCACAGAGAGGT	576-595
HAV-671R	CTTGTTTGGACATATTCATTA	651-671
HAV-732F	TAATGAATATGTCCAGACAAGGT	732-754
HAV-796R	GACAGGATGTGGTCAAGGC	778-796
HAV-855R	CCAGTCACTGCAGTMCTATCAAC	833-855
HAV-923F	GAACCTTTGAAAACCTCTGTTG	923-944
HAV-1093R	AACCTTGAACAGCAAACTGC	1074-1093
HAV-1426F	TTTACAGATTTGGAGTTGC	1426-1444
HAV-1485R	AATTCATTTCTCATCATCTGTGT	1463-1485
HAV-1528F	TATGAAGATGCAAGGGCAAA	1528-1547
HAV-1588F	CTCTCAAGGTGGTGGAATTAA	1588-1608
HAV-1680R	GAATCTGAAGCATTAAATGGA	1660-1680
HAV-1818F	TTGATTTYCAGGTTTTTCC	1818-1836
HAV-1998R	ATCCAAGGAACACGAAATCT	1979-1998
HAV-2120R	AAGCAACATTAGAAGGAGAAGTC	2098-2120
HAV-2210F	GGAGATGATTCAGGAGGTTTT	2210-2230
HAV-2230R	AAAACCTCCTGAATCATCTCC	2210-2230
HAV-2379R	GGATCYTCAATTGTTGTAATAGC	2357-2379
HAV-2412F	CTGAATTGAARCCTGGAGAGT	2412-2432
HAV-2578R	TGGCAAACCATGAGGAGGATTAG	2556-2578
HAV-2746F	TTACAAAACTGCCCTTGGA	2746-2764
HAV-2782F	AAGAAGAACAGGRAACATTCAGAT	2782-2805
HAV-2894F	TCTATTCAGATTGCAAATTACAA	2894-2916
HAV-2921R	AATGATTGTAATTTGCAATCT	2901-2921
HAV-3197R	CATTTGACAACTCTTCCTGAGC	3176-3197
HAV-3263F	ACTGAGGAGCATGAAATAATGAA	3263-3285

HAV-3285R	TTCATTATTTCATGCTCCTC	3266-3285
HAV-3354R	CCAGCAGCCAAAGARAATCCA	3334-3354
HAV-3368F	ACTCTTGAGATGGATGCTG	3368-3386
HAV-3428F	TGGACAGAAATGAARGATGA	3428-3447
HAV-3510F	CACATGGAATGTTGGATCTT	3510-3529
HAV-3581F	TTGTGTTTCYTGTTGCATTG	3581-3600
HAV-3591F	TGTTGCATTGGCTAAATCC	3591-3609
HAV-3602R	GCCAATGCAACARGAAACA	3584-3602
HAV-3911R	AGATAACTGAGCAGCCAAT	3893-3911
HAV-3977R	TCATTCTGGARTCCATTTG	3959-3977
HAV-4103F	AATTATGGCAADAAGAAGGAT	4103-4123
HAV-4179R	GCTTCCTCAATYGCTTTCTCTAT	4157-4179
HAV-4260F	TGAGAACTGTYCAYTCAATGG	4260-4280
HAV-4394F	TGTGAGCCAGTTGTTTGCT	4394-4412
HAV-4413R	TAGCAAACAACTGGCTCAC	4395-4413
HAV-4499R	CCGGTTCAACACCATAGTG	4481-4499
HAV-4567F	CATCATTGATGATATTGGCC	4567-4586
HAV-4636F	ATGCCCAATGAGATTGAATA	4636-4655
HAV-4763R	CAATKGCTTCCTTAACATAAACT	4741-4763
HAV-4770R	CTACGATCAATTGCTTCCT	4752-4770
HAV-4846F	GGCTAAAACAAATGATGCAATT	4846-4867
HAV-4858F	TGATGCAATTAAGGACATGTC	4858-4878
HAV-4917R	AATGAAACATTATGTCCATCCAT	4895-4917
HAV-5007F	CAGATGATGAYAATGATAGTGC	5007-5028
HAV-5240R	TCACGCCATGGTAAACCCC	5222-5240
HAV-5390R	CTCCTAAGGCATTCATAACCC	5370-5390
HAV-5583R	TCTCTAAACTTDGGAATTGTAGGA	5560-5583
HAV-5619F	CTAGAGCTTTGAATCGKYTGGC	5619-5638
HAV-5674F	GGAGATGTACCTAGAGCCTTG	5674-5694
HAV-5788F	AGGTCTTCCWGGAATGTGTG	5788-5807
HAV-5818R	CAAGGCCCCACCACACATT	5800-5818
HAV-6082F	AGTCCCATTCATCATCACATTG	6082-6103
HAV-6364R	GGACATAAGGAAACCCAGG	6346-6364
HAV-6560R	AACTAATAGCTGGACCCCAATAC	6538-6560
HAV-6674R	ATGCCAATAGCAACACCTGT	6655-6674
HAV-6678F	ATTTTTCTGCYTTTGATGCTAGT	6678-6700
HAV-6770F	CAATGCCTTCTGGGTCTCCT	6770-6789
HAV-6792R	ATAGTRTTGATAAGAGCYGTTCC	6770-6792
HAV-6836F	TGTGGTTCAATGCCTTCTG	6836-6854
HAV-7027F	GTGCCTCAATTGAAGCCAGT	7027-7046
HAV-7278R	TGCATAAAAGCAAACCACT	7260-7278
HAV-7477R	ATTTACTGAWAARARAAATAAACA	7454-7477

F, forward direction; R, reverse direction
