# Supplementary material for: Molecular diagnosis of patients with hepatitis A virus infection using amplicon-based nanopore sequencing
Source: PLoS One. 2023 Jul 12;18(7):e0288361. doi: 10.1371/journal.pone.0288361 (PMC10337952; doi:10.1371/journal.pone.0288361)
Supplement: S3 Table — (PDF) [file pone.0288361.s004.pdf]

S3 Table. Genome coverage rate and mean depth for hepatitis A virus (HAV) using nanopore sequencing in the multiplex assay for 24 h.

HAV RNA copy number (copies/µL)	Sample	Type	Coverage rate (%) [a]	Total reads	Reads mapped to reference sequence [a]	Rate of reads mapped	Depth of coverage [b]
10^4-10^5	KUMC 20-4	Stool	82.6	77,450	45,829	59.2	2,060
10^2-10^3	KUMC 20-2	Serum	71.9	115,353	36,036	31.2	1,712
	KUMC 19-1	Serum	71.8	65,102	17,018	26.1	747
	KUMC 20-3	Stool	76.2	46,002	19,457	42.3	937
	KUMC 20-5	Stool	68.3	91,867	16,407	17.9	786
10^1-10^2	KUMC 20-1	Serum	49.8	83,961	2,664	3.2	140
	KUMC 19-1	Stool	43.4	144,904	3,462	2.4	187
	KUMC 20-1	Stool	34.3	126,580	2,611	2.1	139

[a] Genome coverage rates and viral reads mapped to a reference sequence were calculated using the LU38 strain.
[b] Depth of coverage was calculated by the number of mapped reads (read length × number of reads matching the reference/reference genome size).
