# Supplementary material for: Molecular diagnosis of patients with hepatitis A virus infection using amplicon-based nanopore sequencing
Source: PLoS One. 2023 Jul 12;18(7):e0288361. doi: 10.1371/journal.pone.0288361 (PMC10337952; doi:10.1371/journal.pone.0288361)
Supplement: S4 Table — (PDF) [file pone.0288361.s005.pdf]

S4 Table. Indel error rate and consensus accuracy of the nanopore sequencing for hepatitis A virus (HAV) compared to Illumina MiSeq platform.

HAV RNA copy number (copies/µL)	Sample	Type	Insertion	Deletion	Indel error rate (%)	Mismatch	Consensus accuracy (%)
10^4-10^5	KUMC 20-4	Stool	0	6	0.1	15	99.66
10^2-10^3	KUMC 20-2	Serum	0	3	0.06	11	99.74
	KUMC 19-1	Serum	0	5	0.09	13	99.66
	KUMC 20-3	Stool	0	4	0.07	17	99.63
	KUMC 20-5	Stool	0	3	0.06	13	99.69
10^1-10^2	KUMC 20-1	Serum	0	2	0.05	6	99.79
	KUMC 19-1	Stool	0	3	0.09	6	99.73
	KUMC 20-1	Stool	0	1	0.04	14	99.41
